# Healthcare system intervention for prevention of birth injuries – process evaluation of self-assessment, peer review, feedback and agreement for change

**DOI:** 10.1186/1472-6963-12-274

**Published:** 2012-08-24

**Authors:** Monica E Nyström, Anna Westerlund, Elisabet Höög, Charlotte Millde-Luthander, Ulf Högberg, Charlotta Grunewald

**Affiliations:** 1 Department of Learning, Informatics, Management and Ethics, Medical Management Centre, Karolinska Institutet, Stockholm, SE, 171 77, Sweden; 2 Department of Public Health and Clinical Medicine, Umeå University, Umeå, SE, 901 87, Sweden; 3 Department of Clinical Science and Education, Södersjukhuset, Karolinska Institutet, Stockholm, SE, 171 77, Sweden; 4 Department of Women's and Children's Health, Uppsala University, Uppsala, SE, 751 85, Sweden

## Abstract

**Background:**

Patient safety is fundamental in high quality healthcare systems but despite an excellent record of perinatal care in Sweden some children still suffer from substandard care and unnecessary birth injuries. Sustainable patient safety improvements assume changes in key actors’ mental models, norms and culture as well as in the tools, design and organisation of work. Interventions positively affecting team mental models on safety issues are a first step to enhancing change. Our purpose was to study a national intervention programme for the prevention of birth injuries with the aim to elucidate how the main interventions of self-assessment, peer review, feedback and written agreement for change affected the teams and their mental model of patient safety, and thereby their readiness for change. Knowledge of relevant considerations before implementing this type of patient safety intervention series could thereby be increased.

**Methods:**

Eighty participants in twenty-seven maternity units were interviewed after the first intervention sequence of the programme. A content analysis using a priori coding was performed in order to relate results to the anticipated outcomes of three basic interventions: self-assessment, peer review and written feedback, and agreement for change.

**Results:**

The self-assessment procedure was valuable and served as a useful tool for elucidating strengths and weaknesses and identifying areas for improvement for a safer delivery in maternity units. The peer-review intervention was appreciated, despite it being of less value when considering the contribution to explicit outcome effects (i.e. new input to team mental models and new suggestions for actions). The feedback report and the mutual agreement on measures for improvements reached when signing the contract seemed exert positive pressures for change.

**Conclusions:**

Our findings are in line with several studies stressing the importance of self-evaluation by encouraging a thorough review of objectives, practices and outcomes for the continuous improvement of an organisation. Even though effects of the peer review were limited, feedback from peers, or other change agents involved, and the support that a clear and well-structured action plan can provide are considered to be two important complements to future self-assessment procedures related to patient safety improvement.

## Background

Evidence-based knowledge is essential for the improvement of patient safety in healthcare and a process aiming at improving the entire organisation, rather than punishing individuals, is more likely to create sustainable changes in patient safety [1]. However, the gap between what we know and what we do in healthcare is steadily increasing [2]. This is despite the fact that the benefits of adopting new knowledge from scientific findings have been emphasized [3]. In order to achieve higher levels of learning and sustainable changes in patient safety, the involvement of both staff and management is crucial. Sustainable changes assume changes in mental models, norms and culture. Change interventions have to be tailored to prevailing conditions for staff and organisations, considering time aspects, resources and the influence of key actors [4]. Additionally, a descriptive model of the intervention, its process and context is necessary if conclusions are to be drawn on results and effects [5]. How interventions for increasing patient safety in healthcare shall be tailored more exactly and what role external parties can play in this process has, nevertheless, yet to be established. However, the first step in any change effort is to create a readiness for change in the present situation. In a national programme for increased patient safety around delivery we will therefore investigate step-by-step intervention and its effect on enhancing readiness for change i.e. creating a mental representation of the present situation and the need for improvement of safety procedures at a team level.

From an international perspective, Sweden has an excellent record of perinatal care. Despite this fact, some children still suffer from unnecessary birth injuries. The exact numbers of affected children are unknown, but in a recent study from our group, substandard care was found during labour in two thirds of the infants born with signs of asphyxia, and in one third of the healthy infants. The conclusion was that the main reasons for substandard care were factors that, in theory, were potentially reducible through educational efforts and increased awareness of risk factors associated with birth asphyxia [6]. A post-intervention study with a similar design aiming at determining the possible effects of efforts made to improve substandard care is currently being performed. In the national intervention programme for safe delivery, the combination of self-assessment, peer review, a small financial incentive and a thorough follow-up is unique for the healthcare sector in Sweden. It is therefore of importance to evaluate the intervention programme to establish its potential effects and usability in other areas and geographical settings.

The multi-professional team working during a delivery involves midwives, obstetricians, and neonatologists. Teams use team mental models (TMM) to develop common knowledge regarding objectives, solutions, alternatives, chains of action, roles and functions, and the relations between them. The better team members can construct and share mental models the better decision-making performance can be expected [7,8]. TMM helps teams formulate collective explanations of and expectations on the task, share problem representation and orientation, facilitate communication and coordination of team activities, and to develop and sustain situational awareness [9]. In order to get well-functioning teams, team members should have knowledge of all relevant combinations of what the system was doing, why it was doing it, and how it did it [10]. Teams can share declarative, procedural and strategic knowledge in the form of equipment, task, team interaction and/or team attribute models [11]. All these aspects are of interest to patient safety during delivery considering the high technology equipment, variety of task procedures, at times intense team interaction, and the important knowledge of the nature of team member’s expertise.

TMM can also be distributed among team members [12] where members have different pieces of information that can be used to collaboratively build a common model. This indicates the benefit of involving multi-professional teams in improvements and, if a new or adjusted TMM supports learning and innovative knowledge development, it also helps the team to be creative [13]. The collective model makes it easier for team members to share information and knowledge, which can produce synergies for teams engaged in problem solving and decision making. Teams should therefore benefit from possessing skills on how to develop shared mental models of their system, task, goals, risks and decision structure, not least in relation to patient safety.

Self-assessment is often used in order to initiate change efforts by investigating the status quo and comparing it to standards and goals. It is a comprehensive, systematic and regular review of an organisation’s results and activities in order to discern strengths and weaknesses and plan for improvement actions to be monitored for progress [14]. Self-assessment provides an opportunity for organisations to establish structured ways of prioritising actions for improvement, creating possibilities for sharing experiences, collecting feedback, and developing work procedures [14]. The use of a structured self-assessment protocol has been shown to encourage continuous measures of improvements, to support a holistic perspective and to improve the understanding of activity [14-16]. Using self-assessment regularly within an organisation can ensure that sound approaches are used and developed [17]. It encourages a culture of continuous improvement and staff can, by taking an active part in the process, gain a broader understanding of the work itself [15]. Participants often describe the sharing of knowledge and experiences as the main benefit or even as the main objective [14]. Used in the right context self-assessment may encourage fundamental reviews of objectives, practices and outcomes.

The product of a self-assessment is usually a report where a qualitative evaluation, often supplemented with statistical data, is scrutinised by an external party. Additional information is sometimes requested in advance or to be made available during a visit by a peer-review team, a visit that usually lasts between one and four days. In this mixed approach a peer-review team attempts to relate what they hear to the self-assessment document. This mixed model has advantages as it can stimulate fundamental self-reflection and aid continuous improvement by exploring the organisations purpose, its areas of effectiveness, and its weaknesses and future opportunities, followed by an open dialogue and helpful feedback [18].

Peer review or inter-professional assessment is the systematic examination and assessment of the performance of a state by other states, with the ultimate goal of helping the reviewed state improve its policy making, adopt best practices and comply with established standards and principles [19]. Peer review has been used in several areas with varying results [20,21]. In Swedish healthcare (i.e. public health) it has been performed by a group of colleagues working in similar areas as the one reviewed [22]. The learning process for reviewers has then a good potential to be used in praxis. In a Danish study it was concluded that external monitoring can never stand alone and will never be able to replace valuable internal quality monitoring [23]. Some attempts have been made by researchers to determine the effectiveness of peer reviews, but the area is, to a large extent, unexplored [24].

In a mixed approach the self-assessment process might enhance the readiness for a peer review intervention and team participants can be more likely to embrace remarks and comments and include such information in their TMM. It is also possible that a team will take further action and immediately make changes. The peer review itself could therefore have a potential to both acknowledge and strengthen or add new information to develop the TMM already established by the team itself.

Thus, our *purpose* was to study a national intervention programme for the prevention of birth injuries more closely from a demarcated perspective. The *aim* was to elucidate how the main interventions, conducted in the given order, affected the teams and their mental model of patient safety improvement during the intervention process. Difficulties, benefits as well as the likeliness of sustainable changes will be discussed. We also aimed to contribute to the knowledge of which relevant considerations ought to be made before implementing this type of patient safety intervention series with self-assessment, peer review, a small financial incentive and a thorough follow-up.

## Methods

The design is a hospital-based interview study of multi-professional teams. Participation in the study was based on informed consent. The study was approved by the Research Ethics Committee at Karolinska Institutet (No. 2010–1603 31–4).

### Setting

The national intervention - Safe Delivery (SD) - constitutes the empirical basis for the study. SD involves all 46 delivery units in Sweden and is conducted by the following professional organisations: the Swedish Society of Obstetrics and Gynaecology, the Neonatal Section of the Paediatric Society and Swedish Association of Midwives. The Swedish County Councils' Mutual Insurance Company contributed with financial and administrative support. The intervention was divided into three sequences with 1/3 of the units included in each. The duration of each unit of the intervention process was 1.5 years. The first sequence started in September 2008 and the last sequence ended in January 2011. Data from self-assessments and auditions were collected for further evaluation and analysis. The first step of the intervention was a self-assessment based on a questionnaire with questions on how patient safety is secured during the process of delivery, from the first contact with the maternity unit until birth and, if necessary, the transport of the neonate to an intensive care unit. The questions were focused on safety for the infant, on how conditions are be provided for different measures of importance and, not least, how it is ensured that these measures are being followed. A selected group of senior obstetricians, neonatologists and midwives that had completed structured education in this area served as reviewers in the second step of the intervention. Supported by written instructions, documents, and continuous contact with the project management the reviewers fulfilled their obligations. The intervention process for each maternity unit included the following steps:

1. General introduction prior to the self-assessment procedure

2. A written, structured protocol for self-assessment

3. Site inspection by reviewers 1–2 months after the completion of the self-assessment report

4. Written report from reviewers after site inspection

5. Written report on agreement between reviewers and management of maternity unit for measures for improvements, one month after site inspection

6. Written report from maternity unit on measures for improvements taken, six months after agreement

7. The reviewers’ evaluation of the report on measurements taken in order to decide on financial incentive provided for the project

### Procedure

The implementation of the SD programme was carried out in three, partly overlapping, sequences with 13–14 maternity units in each 1½-year long sequence. Sequence I started in September 2008, sequence II in January 2009 and sequence III in September 2009. Representatives from the SD steering committee visited the maternity units, gave a structured introduction and presented the local procedures for unit managers and staff representatives, including neonatologists and anaesthesiologists. The involvement of all professional categories was considered a prerequisite for the self-assessment process. Senior obstetricians, midwives and neonatologist were carefully selected and asked to serve as reviewers by the professional organisations. They obtained detailed instructions on their role and on the peer-review process at a two-day seminar two to three months after the introduction at the maternity unit. At the seminar, peer-review teams of three persons for each maternity unit were formed, each with one representative from each profession. The maternity units’ completed self-assessment reports were distributed and the review process started.

The initial period of sequence I and II was studied, with the main interventions as follows:

A) >*Structured Self-assessment.* The self-assessment survey on safety routines contained 26 questions connected to previously identified risk areas of importance for the prevention of serious birth injuries*.* The areas covered were: Organisation, Communication, Competence, Technique, Handling of prescribed drugs, Documentation and Follow-up. Within each of these areas two main questions were asked:

1) >How do you provide conditions and measures for risk assessment and improvement (including routines, guidelines, equipment, working conditions, etc.)?

2) >How do you ensure that these measures are being followed?

B) >*Peer review.* The maternity units were visited by a peer-review team (see above) two to three months after the initiation of the process. A document based on the questions used in the self-assessment procedure served as support for a structured process. In this document new or updated information found during the visit or in dialogue with the clinic was noted.

C) >The peer-review team presented a *written feedback report* regarding their view on strengths and weaknesses, prioritised areas for improvement and proposed measures to be taken. Ultimately, a *written contract* consisting of a detailed plan of actions was produced in joint agreement by the maternity unit team and the peer-review team.

Based on experiences from sequence I, some minor changes were made for sequence II. The main revisions concerned minor changes in the instructions for the peer-review group, simplifying the protocol used for feedback and a call for a more modest approach during visits (i.e. not anticipating that all clinics had the same basic premises). Each team of peer reviewers during sequence II included a person who had experience from sequence I.

### Participants

For sequences I and II, three respondents per maternity unit were asked to participate in an interview; the management director and two other key team members, usually the head midwife and a senior consultant in obstetrics. In sequence I with14 participating units, we reached all 43 eligible respondents (32 women, 11 men). In sequence II, with 13 participating clinics, we reached thirty-eight of thirty-nine potential respondents (31 women, 7 men). All management directors, except for one in sequence I and two in sequence II participated in the study. In total 80 of 81 respondents were interviewed (99%).

### Interviews

Semi-structured telephone interviews were performed by two interviewers (E.H., A.W.). The same questions were asked to examine each intervention i.e. how the process was perceived, the immediate impact and the intervention’s effects. The interviews lasted for between 15 and 30 minutes. The following questions were asked:

1. How did you, as well as other professionals and staff at your unit, *perceive the process* of conducting the self-assessment/the peer review (visit)/the written feedback report and the signing of the contract (interventions A, B, C)?

2. Did the process of conducting interventions A, B, and C *have any kind of direct impact* at the maternity unit?

3. Did you see any *effects* of the process of conducting interventions A, B, and C?

4. Do you have any other reflections regarding the process of conducting interventions A, B, and C?

The interview manual also contained two additional questions:

5. Are there currently any other patient safety or developmental processes ongoing within your unit that in some way affect or compete with this project?

6. Do you have anything you would like to add?

### Data analyses

#### Content analysis

We (AW, EH, MN) conducted a content analysis with explicit rules for coding. Each interview was transcribed and each segment of text that contained a coherent statement of content (i.e. consistent in content and focus) was separated, coded and categorised in mutually exclusive and exhaustive categories, i.e. sampling units [25]. We applied a deductive analysis using *a priori* coding [26]. Thus, we established the categories prior to the analysis based on theories of mental models, self-assessment and peer review as outlined above. The categories were discussed, refined and agreed upon by the colleagues throughout the process of coding and labelling of text segments.

**Figure 1:**
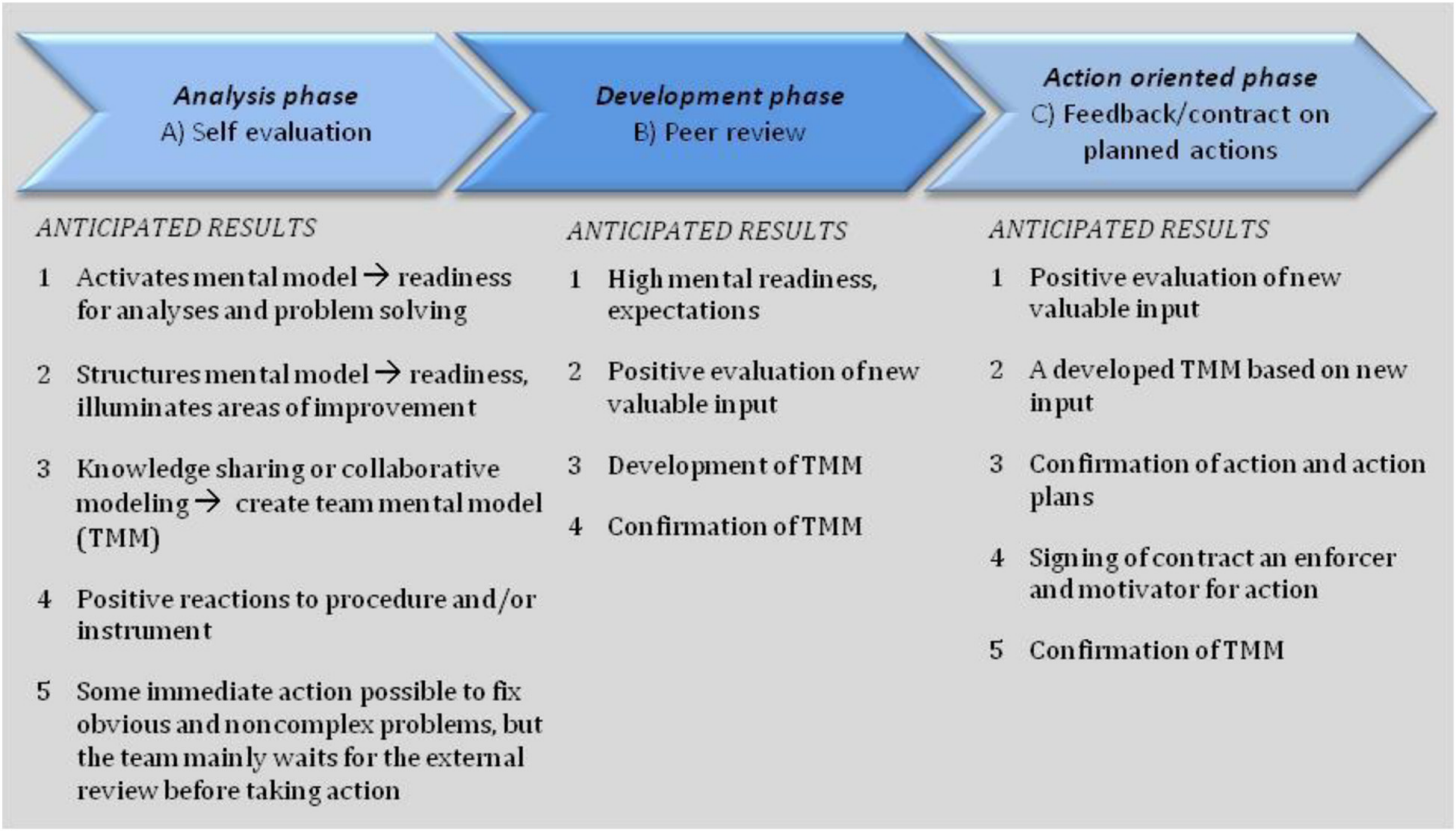
The intervention phases and the anticipated outcomes of each phase in relation to development of TMM and readiness for action.

In Table 1 the rules applied for labelling segments with codes are described for all three steps of the analysis. In the *first step,* text segments were labelled based on *Type of Intervention* (A-C) and in the *second step* the same segments were labelled with a *Model (or effect) code* (M1-8). The Model code specifies the type of effects on a mental or practical level. Segments with no information about effects on a mental or practical level were labelled M0 (e.g. segments simply stating something as positive or negative with no further clarification). The model codes were connected to anticipated outcomes in the intervention series, i.e. the phases of development according to an ideal model (Figure 1). In the analysis phase the structured self-assessment intervention (A) triggers the anticipated outcomes of *activating* (M1) or *structuring* (M2) the TMM, *collaboratively sharing knowledge* (M3), and *taking (minor) actions* (M4). In the development and action oriented phases (Figure 1) the review panel visits (B), the written feedback/discussion, and the signing of the contract (C) are of a more social nature, open for spontaneous input and the adding of values. This can have a *potentially strengthening* effect (M5), be *developing* (M6) or have a *disturbing effect* (M7) on the TMM construction process. As with intervention A, the interventions B and C could result in a team taking further actions and/or result in a written agreement/contract *clarifying measures decided upon* (M8). In a *third step* the segments containing a valuation, were coded as *positive* (a1) or *negative* (a2)

**Table 1:** Step 1–3 in the categorisation and coding process

***Step 1 - Which intervention is the text segment referring to?***	**Code**
Self assessment	A
Peer review	B
Written feedback/signing contract	C
*Step 2: Is the text segment describing an effect? - Mental or practical?*	
*2a - Modelling categories A.*	
Activating mental models	M1
Structuring mental models, illuminating areas of improvement (and strengths)	M2
Knowledge sharing and collaborative modelling – towards TMM	M3
Taking action (practical effect)	M4
*2b- Modelling categories B and C*	
Strengthening/confirming already established TMM	M5
Developing/adding to TMM	M6
Disturbing establishment of TMM	M7
Signing contract and/or taking further action	M8
*Step 3 - Is the text segment a valuation of an experience?*	
Positive	a1
Negative	a2

The result of the procedure provides each segment of text that contains a coherent statement with a *total code* – for intervention A the combination of A – M1-4 a1, a2 or for interventions B and C a combination of B-C M5-8 a1, a2. In the case of the unit simply consisting of a valuation, the total code is M0 a1, a2. All parts of the text that did not fit these definitions were placed in category (O) and analysed separately in order to find general comments of importance.

#### Coding example

The following statement provides an example of the procedure and the final code.

“The process of self-assessment was appreciated, because it made us think deeper, and we found that where we thought we had explicit routines, we actually did not!”

The segment concerns intervention *A* Self-assessment, where the respondent describes an effect on the mental level. The segment is interpreted as referring to “structuring mental models” *M2* and holds an explicit positive value so code a1 is added. The total code is *AM2a1*. Text segments were labelled with a code based on content, regardless of where in the interview it was found.

#### Model used for interpretation

The approach hypothesises that a certain pattern of text segments is desirable for the development of TMM and a readiness for change in each phase of the intervention series. During the analyses the ideal pattern of preferable segments was used as a base for calculations of the proportion of respondents in accordance to the ideal case, as illustrated in Figure 1. The outcomes contained indications of both preferable and absent segments and divergences. This provides a hypothetical pattern for how each intervention should have influenced respondents in order to both achieve a shared TMM and activate actions that promote a successful patient safety system within perinatal care.

#### Analyses of variation in relation to the ideal case

The text segments that contained information related to the three interventions A-C were analysed and compared to the anticipated results, focusing on the ideal pattern when calculating proportion of agreement. The anticipated outcomes for the three basic interventions are summarised in Table 2.

**Table 2:** An overview of the anticipated pattern of text segments for intervention a-c

**The anticipated pattern should:**	**Intervention A**		**Intervention B**		**Intervention C**	
	**Code**	**Description**	**Code**	**Description**	**Code**	**Description**
mainly contain	M1-3	Descriptions of effects on mental level	M6	New input dev-eloped/ added to the team mental model	M8	Team taking further action
	M1-3 a1	Positive valuation of procedure/activities, process/instrument as facilitating the activating/structuring of TMM.	M6a1	Positive valuations of new useful input or idea	M8a1	Positive valuation of action or signed contract/action plan
					M6	New input developed /added to TMM
					M6a1	Positive valuations of new useful input/ idea
partly contain	M4	Minor corrective action taken for non-complex problems illuminated	M5	Peer-review panel confirming own findings (already established TMM)	M5	Written feedback confirming own findings (already established TMM)
	M0a1	General positive valuation	M5a1	Positive valuation of positive/ confirming /strengthening feedback from the review panel	M5a1	Positive valuation of the written feedback containing positive/ strengthening/ confirming info
			M8	Team taking further action	M0a1	General positive valuation
			M0a1	General positive valuation		
not/to minor extent contain	M1-3 a2	Negative valuation connected to mental effects	M5a2	Review panel overly acknowledging own findings (no new input)	M5a2	Written overly acknowledging own findings (no new input)
	M4a2	Negative valuation of actions being taken	M6a2	Negative valuation of new useful input	M6a2	Negative valuation of new useful input
	M0a2	General negative valuation	M7a2	Peer review having a disturbing/disrupting effect on the building of TMM	M7a2	Written feedback having a disturbing/ disrupting effect on the building of TMM
			M8a2	Negative valuation of actions being taken	M8a2	Negative valuation of actions being taken or signed action plan
			M0a2	General negative valuation	M0a2	General negative valuation

Divergences from the ideal case pattern were also analysed. They can hypothetically be of two kinds. The first kind of divergence has a positive or neutral substance, e.g. interventions triggering quick actions during the analysis phase or positive valuations not referring to effects (i.e. with no M-code). These divergences are “harmless”, but should *not* dominate the content. The second kind is less desirable as it concerns a direct or explicitly negative tone or indications of disruption of the construction of a TMM.

To address the proportion of correspondence between the respondents’ results and the ideal case (i.e. the codes corresponding to development of a TMM or taking practical patient safety actions) several analyses were carried out. First, the number of corresponding text segments per respondent was divided by the total number of segments per respondent. Secondly, the number of corresponding segments for the ideal model per intervention (A-C) was divided by the total number of segments per intervention. Thus, the first analysis indicates how many of the respondent’s answers that were more or less similar to the anticipated outcome pattern, based on each individual case. This provides a pattern based on the respondents as units of analysis. In contrast, the second analysis, which is based on the text for all participants in each sequence, does not take into account the individual variation in number of segments. The two analyses together provide a more comprehensive representation of the results. An overview of the maternity unit patterns for each intervention completed the representation of variation.

## Results

The results are presented separately for each intervention, where the coded results are compared with the anticipated patterns. In Table 3 the resulting patterns for all respondents regarding interventions A-C are presented using the segments of text as unit of analysis. In Table 4 the respondents’ patterns are compared with the anticipated outcome pattern, using the respondents as the unit of analysis for interventions A-C.

**Table 3:** Percentage of segments in the model effect (M) and value (a) categories for intervention A-C

	**M**		**Ma1 (positive value)**		**Ma2 (negative value)**	
**A) Self assessment**	Sequence I^*^n = 345%	Sequence II^**^n = 327%	**Sequence I n = 345%**	**Sequence II n = 327%**	**Sequence I n = 345%**	**Sequence II n = 327%**
M1- Activates mental models	10,1	11	3	5,6	0	0
M2- Structuring mental models	24	25	4,2	4,9	0	0
M3- Collaborative modelling (building TMM)	10,1	13	10	4,9	0	0
M4- Taking (minor corrective) actions	14,1	14,4	0	0	0	0
M0- General evaluation	-	-	18,6	12,5	5,9	8,7
**B) Peer review**						
M5- Strengthens/confirms TMM	1,8	5,9	16,3	21	2,1	0
M6- Adding to/develops TMM	6,3	8	10	8	0	0
M7- Disrupting establishment of TMM	0	0	0	0	6,6	2,7
M8- Taking (minor corrective) actions	8,4	8,3	0	0	0	0
M0- General evaluation	-	-	38,5	38,5	10	6,6
**C) Written feedback/Signing contract/ Planning action**						
M5- Strengthens/confirms TMM	1	4,8	11,9	16,6	1	0
M6- Adding to/develops TMM	5,9	5,8	5,5	3,2	0	0
M7- Disrupting establishment of TMM	0	0	0	0	8	8,4
M8- Signing contract/Planning actions	18,4	31,5	8,5	12,7	0	0
M0- General evaluation	-	-	27,8	12,3	12	4,7

* 42 respondents ** 38 respondents.

**Table 4:** Respondents (n) reaching a certain/given proportion of segments matching the ideal case for interventions A-C

**A) Self assessment*Ideal case:*M1-3 or M1-3a1**	**Sequence I (n = 42)**	**Sequence II (n = 38)**	**TOT% (n = 80)**
≥70% of segments correspond with ideal case	9	16	31
≥50% to <70% of segments correspond with ideal case	26	19	57
≥20% to <50% of segments correspond with ideal case	6	2	10
< 20% of segments correspond with ideal case	1	1	2,5
**B) Peer review***Ideal case: M5-8 or M5-8a1*			
≥70% of segments correspond with ideal case	0	2	2,5
≥50% to <70% of segments correspond with ideal case	3	2	6
≥20% to <50% of segments correspond with ideal case	16	10	32,5
< 20% of segments correspond with ideal case	23	24	59
**C) Written feedback/Signing contract/ Planning action***Ideal case: M5-8 or M5-8a1*			
≥70% of segments correspond with ideal case	0	8	10
≥50% to <70% the segments correspond with ideal case	13	11	30
≥20% to <50% the segments correspond with ideal case	18	11	36
< 20% of segments correspond with ideal case	11	8	24

### Self-assessment intervention

For sequences I and II, 61.4% and 64.4%, respectively, of the content of the segments were in line with the ideal pattern (Table 3). For sequence I, 32.7% and for sequence II, 26.9% of the content were slightly divergent, but of non-negative character. For sequence I, 5.9% and for sequence II, 8.7% were content divergent and of a directly negative character.

The resulting pattern of respondents (Table 4) corresponds to a significant extent with the ideal case. Thirty-five of the 42 respondents in sequence I (83%) and 36 of the 38 respondents in sequence II (94.7%) had patterns that matched at least 50% of the ideal case pattern. Thirty-one percent of all respondents (25 individuals) had patterns that corresponded 70% or more, while eighty-eight percent of all respondents’ had patterns that corresponded 50% or more with the anticipated outcome pattern.

The self-assessment process resulted in illuminated areas for improvements but showed also the strengths that the team or the maternity unit possessed. New structures of knowledge and a mutual reference i.e. a *team mental model* emerged during the process and became more or less internalised by the participants. Many respondents indicated that their maternity units already had taken minor action to correct non-complex problems during this first intervention phase. Most respondents expressed positive experiences providing new insights. Examples of the respondents quotations used to describe their experiences of the self-assessment process, with both effects (M1-M4) and evaluations (a1, a2) are provided below.

“The self-evaluation process gave us a chance to elucidate what is really being done and not done”.

“It was very positive to listen to other professionals’ perspectives on different procedures”.

“Both strengths and weaknesses were exposed as we worked through the self-assessment protocol”

“It started an ongoing discussion, and a process where we were all thinking about exactly what we are doing and why as we perform our day to day activities”

“We closely examined every routine and together we made sure the routines were updated and that everyone knew about them”

“Inter-professional participation was absolutely necessary to gain a full perspective on different issues”

“It took time from the regular running of things”

“Difficult to understand some questions, and what answers were expected”

“I would say there was low participation and a lack of interest”

### Peer review intervention

For sequence I, 16.3% and for sequence II, 16% of the content of text segments were in accordance with the ideal case pattern (Table 3). For sequence I, 65% and sequence II, 73.7% of the segments’ content were slightly divergent, but of non-negative character. For sequence I, 18.7% and sequence II, 9.3% was divergent of a directly negative character.

The resulting pattern of most respondents did not match the ideal case (Table 4). Less than 9% of the respondents’ patterns corresponded 50% or more with the ideal case pattern while most respondent’s patterns (59%) corresponded less than 20%.

The intervention was very much appreciated and welcomed by the majority of the participants. The respondents’ valuation of this phase was mainly positive and the visits by the peer panel were viewed as enjoyable and exciting without the feeling of being investigated. It was described as an exchange of knowledge and experiences and gave encouraging feedback and confirmed the teams’ ideas. However, the descriptions of the peer review contained positive valuations without any explicit connection to effects of any kind. This high degree of positive valuations (38.5% of all text segments) implies a significant preparedness *for the peer review but a limited effect on TMM.* The results indicated that the peer-review panel strengthened and confirmed the participants own conceptions, rather than adding new ones – especially in sequence II. The respondents also expressed some negative views and less positive experiences were expressed concerning this phase than for the self-assessment. Based on the amount of positive and negative comments, peer review seemed to be more appreciated in sequence II than in sequence I. The negative judgments mainly concerned suggestions about impracticable measures which were sometimes perceived as very disturbing. In most cases the peer review did not provide much new input that led to further action. The segments indicated ongoing activities initiated as a result of the self-assessment (A). Examples of the respondents’ quotations on the peer-review process with effects (M5-M8) and evaluations (a1, a2) are described below.

“The panel encouraged us to explain ourselves further, which made the picture even clearer for everyone.”

“A review of this kind is great, because it prevents us from becoming blind to defects in our work”

“I cannot say that the peer review brought something new to the table, but it strengthened us in our findings and considerations.”

“They confirmed our ideas and gave the on-going activities we started during the self-assessment an extra push.”

“They used a “big-city” perspective on our little clinic, which was a bit disturbing”

“Some of the proposed measures were totally impracticable. We cannot be expected to rebuild the hospital”

“It felt like they suggested things, just to suggest things. The suggestions were not relevant. They couldn’t come up with anything we hadn’t already thought of.”

“The main reason why we changed routines, and what really got us started, was the self-assessment.”

### Intervention of written feedback, contract negotiation and signing

For sequence I, 38.3% and for sequence II, 53.2% of the text segments were in accordance with the ideal pattern (M8, M8a1, M6, M6a1), while 40.7% and 33.7% respectively, showed divergence of a non-negative character (M5, M5a1, a1). A negative divergent pattern (M5a2, M7a2, a2) was found in 21% of the segment patterns in sequence I and 13.1% in sequence II (Table 3).

The resulting pattern did, to some degree, correspond with the ideal case. Forty percent of all respondents had a pattern that matched 50% or more of the ideal case pattern while 24% of the respondents had patterns that corresponded less than 20% with the ideal case (Table 4).

The content partly corresponded with the ideal case. The respondents’ valuation of this phase was mainly positive, but contained some negative views. Results indicated that receiving a written feedback report, discussing it and signing contracts was very much appreciated by most of the participants in sequence II. The written report strengthened and confirmed established conceptions. The contract, if signed, provided motivation for action. There were indications that some teams at maternity units had already acted on the measures decided upon. The written feedback was delayed (i.e. more than four weeks after the visit), or had been very recently received in seven of fourteen maternity units in sequence I. Thus the feed-back process took longer to execute in Sequence I than in II. Interviews with respondents from these units showed disappointment, obscurity, loss of focus and motivation. This reaction slowed down the process, and in some cases it was perceived as very disturbing. Below are examples of quotations from the respondents describing their experiences of the written feedback/ contract signing process, with effects (M5-M8) and evaluations (a1, a2).

“The written feedback report held no surprises – we have reached an agreement and are currently acting on the measures decided upon.”

“The written report and contract is great because it is a reminder of the measures decided upon”

“The fact that it is written down as a form of decision or contract makes it stronger than if we had just said we were going to do things.”

“The report strengthened and confirmed assumptions and decisions made earlier in the process”

“The report works as a decision support document, sometimes also used at the managerial level”

“The signing of the contract really pushed actions ahead”

“Some suggestions in the report are totally impractical”

“The report provided too little new input. I was expecting more”

### Maternity units’ pattern

The response pattern on organisational unit level were analysed in order to identify maternity units with deviant patterns. In Table 5 the patterns of the maternity units related to intervention A-C in both sequences is presented.

**Table 5:** The anticipated and deviant patterns of maternity units expressed in percentage of text segments

	**A) Self assessment (%)**			**B Peer review (%)**			**C Written feedback, signing contract, planning action (%)**		
	**Ideal**	**Neutral**	**Negative**	**Ideal**	**Neutral**	**Negative**	**Ideal**	**Neutral**	**Negative**
*Maternity units - Sequence I (n = 14)*									
I-1	63	34	3	9	78	13	0	12	88
I-2	58	42	0	9	77	14	30	60	10
I-3	62	38	0	19	70	11	42	58	0
I-4	48,5	48,5	3	10	90	0	43	43	14
I-5	58	42	0	43	57	0	50	45	5
I-6	58	42	0	20	67	13	0	43	57
I-7	60	36	4	0	59	41	13	56	31
I-8	50	50	0	24	70	6	50	50	0
I-9	75	25	0	25	55	20	41	47	12
I-10	47	43	10	25	69	6	46	36	18
I-11	50	19	31	26	48	26	54	31	15
I-12	48	35	17	8	25	67	38	29	33
I-13	86	9	5	15	85	0	36	50	14
I-14	53	47	0	10,5	79	10,5	33	56	11
Range	47-86	9-50	0-31	0-43	25-90	0-67	0-54	12-60	0-88
Mean	58,3	36,5	5,2	17,4	66,4	16,25	34	44	22
SD	11,0	11,7	8,9	10,9	16,8	18,4	17,6	13,3	24,2
*Maternity units - Sequence II (n = 13)*									
II-15	70,5	22	7,5	13,5	73	13,5	69	15,5	15,5
II-16	61	26	13	0	100	0	44	56	0
II-17	67	29	4	29	62	9	50	29	21
II-18	72	9	19	14	86	0	71,5	21,5	7
II-19	59	38	3	25	67	8	33	40	27
II-20	70	27	3	20	67	13	36,5	27	36,5
II-21	62	32	6	0	82	18	61	22	17
II-22	58	39	3	40	60	0	74	26	0
II-23	60	40	0	42	58	0	38,5	38,5	23
II-24	75	25	0	7	93	0	58,5	33	8,5
II-25	53	36	11	7	80	13	34,5	34,5	31
II-26	59	26	15	18	64	18	56	22	22
II-27	42	37	21	17	83	0	53	47	0
Range	42-75	9-40	0-21	0-42	58-100	0-18	33-74	15,5-56	0-36,5
Mean	62,2	29,7	8,1	17,9	75	7,1	52,3	31,7	16
SD	8,9	8,7	7,0	13,4	13,4	7,4	14,3	11,4	12,2

#### Self-assessment

All 27 maternity units had their largest amount of text segments within the ideal pattern for intervention A (range 42-86%). These figures were higher for the maternity units participating in Sequence I (47-86%) than II (42-75%). Two maternity units in Sequence I expressed negative aspects of the self-assessment in more than 10% of the text segments (17-31%) and five in Sequence II (13-21%).

#### Peer review

No maternity unit had their largest amount of text segments within the ideal pattern for intervention B (range 0-43%). These figures were lower for the maternity units participating in Sequence I (0-19%) than II (0-42%), More negative expressions were found for the peer review intervention for nine maternity units in Sequence I (11-67%) and five in II. They expressed negative aspects of the intervention in more than 10% of the text segments related to intervention B (13-18%). The differences between Sequence I and II might be related to the changed instructions to peers between sequences.

#### Written feedback, signing of contract, planning of action

Fifteen of the 27 maternity units had their largest amount of classified text segments within the ideal pattern for intervention C, four in Sequence I (range 0-54%) and eleven in II (range 33-74%). Ten maternity units in Sequence I (11-88%) and eight in II (15.5-31%) expressed negative aspects of intervention C in more than 10% of the text segments. The differences between maternity units in Sequence I and II might be related to the delay in this intervention for some units in Sequence I.

### Other findings

Text segments that could not be coded in accordance with intervention (A-C) and model code (M0-8) were placed in category (O). From this category very general comments regarding the entire SD programme and its potential future development were separated and summarised. These comments show that most respondents sum up the project as being very positive and highly appreciated. The fact that the project was initiated by professional organisations is noted as very beneficial. The few explicitly negative comments relate to the questioning of working with safety issues in a project format and the sustainability of the effects. Three main issues frequently arose in the interviews: 1) suggestions or requests to share knowledge about procedures and solutions across maternity units (general outlines or guiding principles nationwide), 2) requests to also include the maternal perspective (maternal safety issues) in this kind of project, and, 3) the view that this type of intervention series ought to be conducted continuously. Another positive side-effect was the dissemination of knowledge that also occurs as the peer reviewers take back valuable impressions and ideas to their own hospitals. Some answers indicated that one would not have done such thorough work with self-assessment without the planned follow-up and visit from a peer review panel – an important aspect to consider when interpreting data on the peer review intervention. No significant interference from other projects was found based on the participants’ answers.

## Discussion

The results of this study indicate that the *self-assessment procedure* is worthwhile and can serve as a useful tool for elucidating strengths and weaknesses and thus identifying areas for improvement for safer deliveries within a maternity unit. The peer review part of the intervention was appreciated by the majority of the participants, despite it being of less value when considering the contribution to explicit outcome effects (i.e. new input to TMM and new suggestions for action). Our findings are in line with several studies that stress the importance of self-evaluation, by encouraging a thorough review of objectives, practices and outcomes, for the continuous improvement of an organisation (Karlsen RaS, B. Between governmental demands and institutional needs: peer discretion in external evaluations—what is it used for? In. paper presented at the 17thAnnual EAIR Forum, Dynamics in Higher Education: Traditions Challenged by New Paradigms, 27–30 August 1995. ed. Zurich, Switzerland; 1995.) [27-30]. Thus, self-assessment could serve as a worthwhile structured process for continuously analysing and prioritising actions to promote patient safety and improve quality of services in healthcare.

Samuelsson and Nilsson (2002) [14] stress the importance of not seeing self-assessment as a separate activity but as part of a holistic perspective on how to structure ways of prioritising actions for improvement, collecting feedback, sharing experiences, and developing work procedures in organisations. Self-assessment can aid in structuring communication and lay the foundation for a TMM if all team members are allowed to participate and contribute. However, as pointed out in an editorial by Davies, getting health professionals to collaborate is about more than simply working side by side [31]. The National Institute for Clinical Excellence has nevertheless acknowledged the importance of sharing knowledge since it is impossible today for individual organisations and teams to stay at the forefront of knowledge given the rapid organisational and clinical change [32].

In order to maintain the highest standard of patient safety at a maternity unit an ideal TMM implies full knowledge of the whole birthing process, including roles and activities other professions are expected to fulfil in different phases and situations. New TMM structures of knowledge and a mutual frame of reference emerged during the intervention process and became more or less internalised by the participants in the study. Moreover, many teams had already taken action to correct minor, non-complex problems during this first phase of the intervention. Thus, the self-assessment process as the first step of the SD intervention was appreciated by the majority of its participants, but concern about its sustainability was often raised. A TMM derived in an open process with committed team members can increase the sustainability of the results and might also aid the creation of a learning culture. If self-assessment is used as a regular tool, the TMM will be continuously updated, especially if new information on risk areas is considered in the process.

It seems likely that the established team mental model and mutual frames of reference lead to enhanced readiness for peer review and also for change. The first step in a change process is often to understand the short-comings of the present situation or action strategies in use [33]. Self-assessment could also facilitate effective implementation of new guidelines and routines. If the self-assessment process is properly used it activates individual’s mental models as part of learning and knowledge structuring.

The effects of *peer review* or inter-professional assessment have been studied less and its use has been debated within medicine [34]. The peer review itself seems to be valuable in strengthening the TMM and encouraging team members to clarify and modify their shared mental model of patient safety procedures at the maternity units. Thereby it increases the range and veracity of the TMM and probably also strengthens motivation for action. For the purpose of identifying areas for improvement, planning, and executing interventions the practical value of the peer-review process seems to be questionable. In general the on-site visit of the reviewer group at the maternity units did not substantially generate new ideas, even though it was regarded as being “nice and supportive”. The importance of instructions, task approach, and training of reviewers also emerged during the progress of the programme.

When designing the SD intervention programme, the hypothetical side-effect of a large number of peer reviewers being selected as opposed to a small one would be the possibility of bringing good ideas for patient safety issues back home. This effect was considered by the steering committee to be more important than the design of a more homogenous intervention, as would have been the case with a limited group of carefully selected reviewers. According to our results, this positive side effect was appreciated although the extent to which it had an impact on patient safety is uncertain.

The *feed-back report* and the mutual agreement on measures of improvements made when *signing the contract* seemed to exert positive pressure for change at the maternity units. Some of the obstacles mentioned were the critical attitudes and unrealistic suggestions made by some of the reviewers. The reviewers were criticised for proposing solutions that were not applicable in small units situated within rural, less-populated areas. This was more evident in Sequence I and resulted in clear instructions for the reviewers involved in Sequence II to be more humble and to have realistic expectations on achievable measures of actions within the prevailing healthcare system. These aspects raises questions not only on how to use the peer-review process in relation to the other interventions, but also how peer reviewers should be selected, trained and instructed.

It remains to be seen how external initiators can provide feedback and exert positive pressure for improvements in effective ways and how they can act in order to enhance sustainability of positive results. The role of the unit managers with regard to this issue is also an open question. The SD project has used a substantial amount of the project’s funding in preparing and carrying out peer reviews on 28 visits during sequence I-II. Maybe there are other alternatives? Nevertheless, self-assessment is often taken more seriously if a peer review is to follow, even though the actual peer reviews are often not a particularly effective or efficient means of finding out what is really going on [35]. Others underline the potential of self-evaluation combined with peer review as being effective in enhancing continuous improvement if outcomes are linked to day-to-day activities [18]. An alternative model often used in Sweden for improving quality in healthcare on a national basis is the *IHI Breakthrough model* focusing on small scale tests of ideas and new approaches [36], using the Plan-Do-Study-Act cycle [37]. It requires the cooperation of organizational units and the formation of improvement teams, learning seminars, change facilitators etc. and might be useful for the continuing development process on each maternity unit, given adequate resources.

An integration of the self-assessment process with overall corporate planning and other follow-up systems is inevitable to guarantee success in the long run. Integration into the organisational system is crucial in order to achieve a well-functioning loop of analyses, planning, change/improvement, follow-up, and evaluation as well as a basis for the continuous improvement of patient safety and quality.

There are some methodological considerations that need to be highlighted. The fact that seven of the fourteen participating maternity units had not fully received or had just recently received the written feedback report and had started negotiating the contract by the time the interview took place in Sequence I must be taken into account when interpreting the results. This was an unexpected delay according to the plan and in three of these units this issue was considered to be very unfavourable and problematic.

The instructions also changed between sequence I and II, partly due to the peer reviewers approaches during their visits, perceived unrealistic peer reviewer demands on the clinics, and the time that passed between the visit and the written feedback, discussions and the signing of contracts. These aspects are examples of changes in longitudinal improvement programmes that make it more difficult to evaluate and compare results over time.

The duration of the interviews was relatively short due to the number of respondents involved and their time constraints. Longer interviews with extended questioning on their experiences of interventions A-C would probably have provided more details on the specific procedures and effects, and on the development process of TMM.

It is importance to point out is that even though frequencies and proportions were used based on the qualitative data, the main purpose was to get a general overview of the response pattern in relation to a hypothetical pattern that would give an idea of the three interventions’ potential impact on TMM and the readiness for taking actions. In other circumstances these figures should be interpreted with caution.

Not included in the study is information on the perspectives of the women giving birth and her partner, which could have provided useful insights and understandings on how to improve patient safety during delivery.

## Conclusions

Our findings are in line with several studies stressing the importance of self-evaluation by encouraging a thorough review of objectives, practices and outcomes, for the continuous improvement of an organisation. Even though the effects of the peer-review were limited, feedback from peers, or other change agents involved, and the support that a clear and well-structured action plan can provide are considered to be two important complements to future self-assessment procedures related to patient safety improvement. For a full evaluation of peer reviews or different combinations of interventions involving self-assessment, peer review and affirmative pressure for action and follow-ups, future studies also need to take into account the peer reviewers themselves and the potential transfer effect to their own units. The most important questions to consider for the future of the SD programme is how to further enhance sustainability of improvements and lessons learned and to consider what role professional organisations can have in patient safety issues in the long run.

## Competing interests

The authors declare that they have no competing interests.

## Authors’ contributions

MN, EH, CG and UH designed the study, EH and AW collected the data, MN, AW and EH conducted the analyses and MN and AW drafted the manuscript. CG, UH and LL made substantial contribution to the manuscript and its conclusions. All authors read, contributed to, and approved the final manuscript.

## Pre-publication history

The pre-publication history for this paper can be accessed here:

http://www.biomedcentral.com/1472-6963/12/274/prepub
